# Predicting dissolution and transformation of inhaled nanoparticles in the lung using abiotic flow cells: The case of barium sulfate

**DOI:** 10.1038/s41598-019-56872-3

**Published:** 2020-01-16

**Authors:** Johannes G. Keller, Uschi M. Graham, Johanna Koltermann-Jülly, Robert Gelein, Lan Ma-Hock, Robert Landsiedel, Martin Wiemann, Günter Oberdörster, Alison Elder, Wendel Wohlleben

**Affiliations:** 1 Department Experimental Toxicology and Ecology and Department Material Physics, BASF SE, 67056 Ludwigshafen, Germany; 2 Institute of Pharmacy, Faculty of Biology, Chemistry & Pharmacy, Freie Universität Berlin, 14195 Berlin, Germany; 3 National Institute of Occupational Safety and Health, Cincinnati, Ohio 45226 USA; 4 Biopharmaceutics and Pharmaceutical Technology, Saarland University, 66123 Saarbrücken, Germany; 5 IBE R&D Institute for Lung Health gGmbH, Mendelstr. 11, 48149 Münster, Germany; 6 University of Rochester Medical Center, Rochester, New York USA

**Keywords:** Nanoscale biophysics, Cell-particle interactions, Methods of toxicology studies

## Abstract

Barium sulfate (BaSO_4_) was considered to be poorly-soluble and of low toxicity, but BaSO_4_ NM-220 showed a surprisingly short retention after intratracheal instillation in rat lungs, and incorporation of Ba within the bones. Here we show that static abiotic dissolution cannot rationalize this result, whereas two dynamic abiotic dissolution systems (one flow-through and one flow-by) indicated 50% dissolution after 5 to 6 days at non-saturating conditions regardless of flow orientation, which is close to the *in vivo* half-time of 9.6 days. Non-equilibrium conditions were thus essential to simulate *in vivo* biodissolution. Instead of shrinking from 32 nm to 23 nm (to match the mass loss to ions), TEM scans of particles retrieved from flow-cells showed an increase to 40 nm. Such transformation suggested either material transport through interfacial contact or Ostwald ripening at super-saturating conditions and was also observed *in vivo* inside macrophages by high-resolution TEM following 12 months inhalation exposure. The abiotic flow cells thus adequately predicted the overall pulmonary biopersistence of the particles that was mediated by non-equilibrium dissolution and recrystallization. The present methodology for dissolution and transformation fills a high priority gap in nanomaterial hazard assessment and is proposed for the implementation of grouping and read-across by dissolution rates.

## Introduction

Knowledge about pulmonary retention kinetics of inhaled particles is an essential element of hazard assessment and of understanding the mechanisms by which adverse health outcomes may occur. Barium sulfate was generally assumed to be poorly-soluble and of low toxicity unless delivered at high concentrations over an extended period^1,2^. However, Konduru and colleagues reported that intratracheally instilled ^131^BaSO_4_ NM-220 exhibited a lung retention half-time of only 9.6 days in rats and that ^131^Ba was incorporated into the bones, suggesting nanoparticle dissolution and/or translocation to extrapulmonary sites^3^. A subsequent 90-day inhalation study in rats with a high concentration of aerosolized BaSO_4_ NM-220 (50 mg/m^3^)^4^ revealed no signs of lung overload and a retention half-time of 56 days, which is close to the normal range for the rat lung^4^. A two-year rat inhalation study with BaSO_4_ NM-220 (50 mg/m^3^), however, demonstrated an increase of retained Ba in the lung during the first year of exposure, after which a steady-state was achieved^5^. Since significant Ba accumulation in bone and bone marrow was also observed and, given that the measurements of Ba distribution [1–3] provide no information about its physicochemical characteristics, the complex *in vivo* dissolution and/or transformation of BaSO_4_ secondary to inhalation exposure require more detailed investigation.

Particle clearance from the lung involves absorptive (dissolution) and non-absorptive (physical) mechanisms. For poorly-soluble particles, physical clearance mechanisms – involving macrophage engulfment, transport, and mucociliary propulsion towards the oropharynx – dominate the pattern of overall clearance. Kreyling^6^ demonstrated a retention half-time of 70 days in the rat for poorly-soluble particles^7^. For metal or metal oxide nanoparticles that undergo *in vivo* dissolution, clearance may not be immediate due to biotransformation and binding events (proteins or other biomolecules) that prolong retention^8–10^. When dissolution starts at the oxidized surface layer of a metallic nanoparticle, there is a continuous process of ion-leaching and oxidation. After the original oxide layer was leached, a second-generation oxide layer forms on the shrinking particle. The binding events may slow down physical transport to compartments of more aggressive acidity, thus also slowing down the onset of dissolution after inhalation. Thus, the collective *in vivo* observations with lung-deposited BaSO_4_ suggest that it is more biosoluble than assumed and, therefore, *in vivo* dissolution and processing must be considered.

The evaluation of particle solubility is a key element of many integrated testing strategies^10–12^ and of frameworks for categorizing broad classes of materials, such as engineered nanomaterials (ENMs), in terms of their physicochemical properties^13–19^. Methods to assess the equilibrium (or quasi-dynamic) solubility of ENMs that are suspended in water or physiological buffers – as was done with BaSO_4_ and supported the conclusion regarding its low solubility – are relatively well developed^20,21^. The OECD draft guideline under current discussion involves suspending particles in a medium, incubation, removal of remaining solids by centrifugation or ultrafiltration, and measurement of the analyte in solution^22^. These approaches could be adequately predictive of particle dissolution in a closed system, e.g., a cell culture well for *in vitro* exposure studies^21^. There are several drawbacks with these approaches, however, when the model in question is an *in vivo* one. First, the lung is not a static (equilibrium) system, as the ions that are liberated from the particles via dissolution are continuously removed from the compartment where deposition originally occurred, or they become bound to biomolecules or may form secondary nanoparticles via reprecipitation. Secondly, if the closed system reaches the solubility limit in the selected medium, the dissolution rate is easily underestimated and particles with some solubility may appear as very poorly-soluble. Thirdly, commonly-used methods generally lack a means for evaluating the structural transformation of remaining solids, i.e., physicochemical modifications that could impact clearance and particle disposition. Lastly, any abiotic system does not fully reflect disposition in lung because the lining and interstitial fluids throughout the respiratory tract are pH-balanced, complex mixtures of salts, serum proteins, and other biomolecules. Most importantly, phagocytosis by macrophages or other cell types introduces particles to the lysosomal microenvironment with an acidic pH. Thus, a dynamic (non-equilibrium) system with more realistic media composition that simulates extra- and intracellular lung environments with both fluid phase and solid phase product analysis may be better suited to an evaluation of the *in vivo* bioprocessing of deposited particles in the lung, particularly if both the intraphagolysosomal and lung surface microenvironments are considered.

Indeed, dynamic systems were developed and validated to estimate the biopersistence of mineral fibers^23–25^. In these systems, dissolved ions pass through a membrane with a pore size that excludes the parent particles. The ions on the other side of the membrane are continually removed from the system using flow-through or flow-by macrodialysis, thus achieving non-equilibrium conditions over the time course – hours to days – of the study. The dialysate is collected in discrete volumes, after which the target analyte is quantitated in the collected fractions and the waste that was not sampled. Adaptation to ENMs mainly requires the choice of appropriate separation membranes. Stefaniak and colleagues employed a membrane to separate the suspended particles from a larger volume of particle-free receptor medium, thus gaining size exclusion in addition to the flow-mediated concentration gradient that provided short-term disruption of equilibrium conditions^21^. Another quasi-dynamic system with relatively large volume was explored and a setup patented for oral exposure purposes^26^, demonstrating that dynamic setups can also be employed to study the structural transformations of remaining solids^27^. Transformation of nanoparticles by *in vivo* processing has been directly observed for the relatively biosoluble amorphous SiO_2_ in the pulmonary compartment^9^. Transition metal oxides, specifically CeO_2_, have also demonstrated the potential to recrystallize in lysosomal conditions^28^ or in extracellular medium^9^, and bioprocessing was observed to be organ-specific^29^. Such modulations of biopersistence by local physiological conditions could, thus, contribute significantly to the unusual biokinetics of nanoscale BaSO_4_^4,5,30^.

We hypothesize that both the shedding of ions and *in vivo* biotransformation of remaining solids contribute to the biokinetics of nanoparticles. We describe here methodology to evaluate the abiotic dissolution of BaSO_4_, thought to be a poorly-soluble ENM, and explore the extent of agreement that can be reached in comparison to *in vivo* results.

## Materials

Previous *in vivo* studies on BaSO_4_ NM-220 have already been conducted^3,4^ and their physicochemical properties published in multiple reports^31–33^. BaSO_4_ NM-220 is a benchmark material of the OECD sponsorship program. Table 1 lists physicochemical properties of NM-220 as relevant for ECHA nanoforms^34^.

**Table 1:** Physicochemical properties of BaSO_4_ NM-220.

Property	BaSO_4_ NM-220
Composition/crystallinity/impurities (XRD*)	purity > 93.8%; Na, Ca, Sr, F, Cl, organic compounds
Minimum external dimension (TEM**)	32 nm
Shape (TEM**)	Spheroidal
Specific surface area (BET***)	41 m²/g
Surface modification	None
Contact angle (water)	<10° (hydrophilic)

^*^X-ray diffraction (XRD); **Transmission electron microscopy (TEM); ***Brunauer-Emmett-Teller method (BET).

## Methods

### Static solubility or quasi-dynamic abiotic dissolution

Details for testing the solubility of BaSO_4_ in phagolysosomal simulant fluid (PSF) under static conditions are provided in the *Supplementary Information*. In short, BaSO_4_ was suspended in 200 mL PSF (composition described in^21^) at a concentration of 10 mg/mL, then incubated for 7 or 28 days at 37 °C with stirring. The remaining particulate matter was separated from the ions in solution using ultracentrifugation at 67,000 × g for 2 h, and the Ba concentration in the supernatant fraction was analyzed by inductively-coupled plasma mass spectrometry (ICP-MS). Particle dissolution under quasi-dynamic conditions was performed by suspending BaSO_4_ in PSF (10 mg/mL) and injecting the suspension into a 2 mL dialysis cassette with a cut-off at 7 kDa (*Supplementary Information)*. The dialysis cassette was placed horizontally in a glass vessel filled with 200 mL PSF as receptor medium at 37 °C with stirring. The receptor medium was exchanged daily and analyzed by ICP-MS. The methodological limit of detection for Ba was 0.1 mg/L.

### Flow-by abiotic dissolution

**Figure 1:**
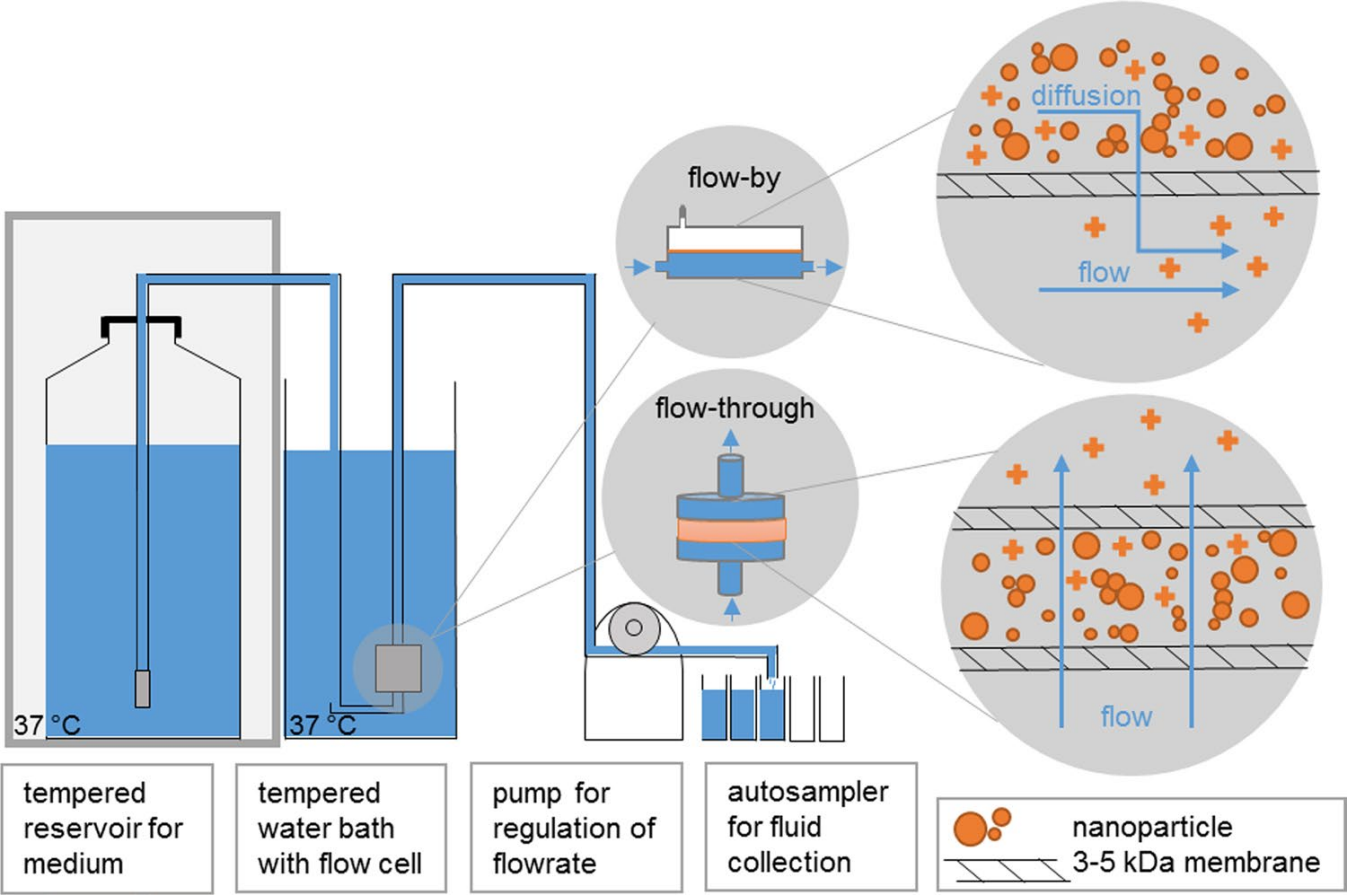
Dissolution method in abiotic flow cells (adapted from^37^). The medium was selected to match the conditions of either the phagolysosomal (pH 4.5) or lung lining fluid (pH 7.4) microenvironment. Particles are in direct contact with the membrane. The choice of the ultrafiltration membrane permeation cutoff is essential; a range 3 kDa to 5 kDa is recommended. This represents a size cut-off of ~1–2 nm (Ren *et al.*, 2006). Smaller cutoffs could induce excess pressure drops and are not recommended. Other options include (recommended): (1) especially for flow-through operation, anti-clogging filters on inlet tubing in the reservoir and elevation of the reservoir by roughly 30 cm, such that hydrostatic pressure compensates for the pressure drop by ultra-high molecular-weight polyethylene (UHMWPE) ultrafiltration membranes. (2) In one implementation, we operated five cells in parallel with a programmable autosampler. Each cell then has its own reservoir inlet tube, peristaltic pump tubing, and sampling.

The setup implements a Continuous Flow System (CFS) according to ISO TR 19057. A dynamic flow-by macrodialysis system^23,24^ has been employed to estimate the *in vivo* dissolution of ENMs^35^. Here (Fig. 1), BaSO_4_ (~1 mg/mL) was suspended in dissolution buffer before being injected into the upper chamber of a dialysis cell fitted with a 3.5 kDa cellulose ester symmetric membrane (Spectra/Pore^®^, Gardena, CA; effective pore size ~1.4 nm). The Ba-free dissolution buffers simulated extracellular lung lining fluid (pH maintained at 7.4 by bubbling 5% carbon dioxide into the buffer reservoir) or intraphagolysosomal fluid (pH adjusted to 4.5 with HCl). The latter of the two buffers is termed EU pH4.5 herein (see composition in *Supplementary Information*). The dialysis cells were submerged in a 37 °C water bath in a dark room. The buffers flowed by the dialysis cells at a rate of 60 μL/min, or ~3 mL/h. A fraction collector with metal-free, pre-weighed polypropylene tubes was used to collect the dialysates over the course of 7 days. For the first 24 h, two-hour fractions were collected (so, 12 fractions for Day 1); thereafter, the fractions were combined such that there were 6 daily fractions. The sample weight for each tube was recorded. After 7 days, the following additional samples were collected in ultra-clean polypropylene digestion tubes for analysis: the remaining solids in the upper chamber; three rinses with 18 MΩ deionized water; and the dialysis membrane. The tubes were placed in a 90 °C heating block. Ultra-pure nitric acid was added to dissolve the membrane and the BaSO_4_ nanoparticles. The Ba remaining in the upper cell after 7 days, the Ba left in the dialysis membrane, and the Ba found in each fraction was quantitated via atomic emission spectroscopy (Beckman Spectraspan V, Fullerton, CA; instrument limit of detection, ~10 µg/L).

### Flow-through abiotic dissolution and transformation

The flow-through setup (Fig. 1) was recently described in detail as another implementation of a CFS according to ISO TR 19057^36,37^. CFS is established as a screening method of the dissolution kinetics of mineral fibers^25,38,39^. Unless otherwise mentioned, an ENM mass of M_0_ = 1 mg was weighed onto a membrane (cellulose triacetate, Sartorius Stedim Biotech GmbH, Goettingen, Germany: 47 mm diameter, 5 kDa pore size), topped by another membrane, and enclosed in flow-through cells. The flow through cells were kept upright within a tempered water bath to ensure that emerging air bubbles can leave the system and do not accumulate within the cell. The initial surface area SA is M_0_*BET (Table 1). The flow rate (V) was 48 mL/d, but was varied up to 100 mL/d. For the lower flow rate, this corresponds to a ratio, SA/V = 0.02 h/cm. The compositions of simulant fluids vary significantly in literature^40^. With the compositions documented in Table S1, the EU pH4.5 medium with a whole range of organic acids, or the simpler PSF medium –previously validated for the purpose of particle dissolution^21^– were employed at 37 ± 0.5 °C. The programmable sampler drew 10 mL eluates once per day from the total 100 mL collected. The Ba concentration in the eluates was determined by ICP optical emission spectrometry (ICP-OES, Agilent 5100). After the experiment, the cells were flushed with deionized water before opening them to rinse the remaining solids off the membrane. The resulting suspension was then pelleted onto a transmission electron microscopy (TEM) grid held at the bottom of a centrifuge vial within 30 min and then dried^37^ so that the morphology of the remaining solids could be inspected with a reduction of interference from drying artifacts of PSF salts, which are removed by this preparation. Particle morphology was analyzed by TEM with a Tecnai G2-F20ST or Tecnai Osiris Microscope (FEI Company, Hillsboro, USA) at an acceleration voltage of 200 keV under bright-field conditions. X-ray photoelectron spectroscopy (XPS) was done using a Phi Versa Probe 5000 spectrometer using monochromatic Al Kα radiation.

### Derivation of dissolution rates

For both of the flow-cell setups, we multiplied the measured Ba concentration of each eluate by the eluted volume to obtain a mass of dissolved Ba ions per sample and then stoichiometrically adjusted this value to obtain the dissolved mass of BaSO_4_ at each sampling interval, Δt. We then analyzed the dissolution kinetics in three alternative ways:

- Cumulative rate: The amount of dissolved BaSO_4_ at each time point M_ion_(T), is expressed as a fraction of the initial mass loading (M_0_ = 100%) and cumulated from all samplings with concentration c_i_, flow V_i_ and sampling interval Δt_i_, and includes the stoichiometry of BaSO_4_:

1a $$\frac{{M}_{ion}(T)}{{M}_{0}}=\frac{m(BaS{O}_{4})}{m(Ba)\ast {M}_{0}}\ast \mathop{\sum }\limits_{i=0}^{T}{c}_{i}(Ba)\ast {V}_{i}\ast \Delta {t}_{i}$$

1b $$k=\frac{{M}_{ion}(T)}{{M}_{0}}\frac{1}{T\ast BET}$$

The rate k incorporates the BET value in order to report results with a focus on composition or coating dependence, instead of size dependence. The conventional units of k are ng/cm²/h^25,41^. We typically determine k by the cumulated ions at the end of the test.

- Curve fitting: To verify first-order dissolution kinetics^41^, the cumulative dissolved BaSO_4_ mass is expressed as an inverse relationship, i.e., decreasing solid retained BaSO_4_ mass (M_ion_(T) − M_0_)/M_0_, and plotted against time on a semi-log scale. The dissolution rate – expressed as a fraction per hour – is calculated from the slope of this line and then converted to percent per day using the total system available starting mass. Dissolution rate and half-time (t′_1/2_, 50% dissolved) are inversely related and can be expressed in two alternative metrics (below) as given for first order modeling in ISO 19057:2017^36,41^. The BaSO_4_ dissolution half-time allows direct extrapolation and comparison to the *in vivo* dissolution t_1/2_ of inhaled BaSO_4_, which is derived from the total *in vivo* t_1/2_:

2a $${b}_{diss}=\frac{\mathrm{ln}\,2}{{t^{\prime} }_{1/2}}\,\,{\rm{or}}\,t{^{\prime} }_{1/2}=\frac{\mathrm{ln}\,2}{{b}_{diss}}$$

2b $${k}_{diss}=\frac{\mathrm{ln}\,2}{{t}_{1/2}\ast BET}\,{\rm{or}}\,{t}_{1/2}=\frac{\mathrm{ln}\,2}{{k}_{diss}\ast BET}$$

- Instantaneous rates: For each sampling interval Δt, the instantaneous dissolution rate k was constructed as:

3 $${\rm{k}}({\rm{t}})={{\rm{M}}}_{{\rm{ion}}}({\rm{t}})/{\rm{SA}}({\rm{t}})/\Delta t.$$

We approximated the instantaneous surface area

4 $${\rm{SA}}({\rm{t}})={\rm{BET}}({\rm{t}}=0)\ast ({{\rm{M}}}_{0}\,-\,{{\rm{M}}}_{{\rm{ion}}}({\rm{t}}))$$

and, thus, ignored changes of the size distribution and shape (see Discussion). Elsewhere^37^ we explore modeling of SA(t) via the assumption of shrinking spheres^36,41^, which does not apply for particles with a tendency to transform, such as BaSO_4_.

It should be noted that the BET value that is used in Eq. (2b) for the determination of t_1/2_ cancels out with BET in Eq. (1b). Accordingly, the two evaluation approaches (fitting vs. cumulated rate) should coincide if the assumption of exponential decay (first order kinetics) is true. Also, the cumulative rate and the instantaneous rate should coincide in the absence of transformation during the test.

Expression of the rate as k_diss_ favors the read-across between nanoform and non-nanoform, because it eliminates the dissimilarity of the specific surface and focuses on modulation of the rate by different coatings or different crystallinities. The expression of the rate as b_diss_ avoids uncertainties regarding changes in particle surface area during the test and indicates a fraction of mass loss per unit time, which can readily be compared to *in vivo* dissolution rates – or to predict them – with the assumption of rapid clearance (no binding) of the dissolved ions.

### Evaluation of *in vivo* structural transformations via high-resolution analytical TEM

Rat lung blocks from the 24-months inhalation study at 50 mg/m³ of BaSO_4_ NM-220^5^ were cut to 50 to 70 nm thick sections and collected on 200 mesh Formvar/carbon coated copper grids. For ultrastructural and elemental characterization of selected lung sections high resolution scanning transmission microscopy (HRSTEM) was performed using a JEOL 2100 F field emission TEM/STEM operated at 200 keV with an analytic pole piece. Tissue sections for HRSTEM were prepared without staining or osmication since OsO_4_ nanoparticles form and can bind to select tissue regions, which makes it difficult to optically distinguish those from potentially inhaled BaSO_4_ nanoparticles or second-generation particles from *in vivo* processing. Images were recorded with a Gatan Ultrascan 4kx 4k CCD camera and data analysis and processing used Gatan Digital Micrograph software (Gatan, Inc.). HRSTEM imaging and Energy-dispersive X-ray spectroscopy (EDS) were performed with a GATAN HAADF detector, Digiscan II, Gatan 2000 Image Filter (GIF), and an Oxford Aztec EDS system (Oxford Instruments, Oxfordshire, United Kingdom) respectively. All HRSTEM images were acquired using an analytical probe with 0.17 nm. A FEI Talos transmission and scanning electron microscope was used for fast EDS mapping with a high degree of sensitivity due to the wrap-around style EDS detector mounted on the objective lens. Maps generally took 1 to 2 min to acquire with a sensitivity great enough to detect elemental concentrations in 4-nm size particles. EDS provides the means to determine the relationship between elemental accumulation and tissue regions, particularly in a situation where dynamic processes may be in play such as *in vivo* processing^9^.

### Ethics approval and consent to participate

All experiments were performed in accordance with relevant guidelines and regulations. The long-term two-year inhalation study with interim sacrificing after 12 months exposure was approved by the local authorizing agency for animal experiments (Landesuntersuchungsamt Koblenz, Germany) as referenced by the approval number G 12-3 028.

### Consent for publication

All authors read and approved the final manuscript.

## Results

### Static abiotic solubility

Data on the water solubility of BaSO_4_ (2.45 ppm Ba ions at 20 °C^42^) cannot explain the *in vivo* observations. Even when solubility was measured in a medium that mimicked the intraphagolysosomal space, BaSO_4_ was classified as insoluble with less than 0.1% dissolved (1000 ppm Ba ions)^3^. Here we replicated the static solubility measurements in different media and found 8 mg/L BaSO_4_ (or 0.08%, nominal) dissolved in pH 4.5 phagolysosomal simulant fluid (PSF) over 7 days, stagnating at 5 mg/L (or 0.05%, nominal) after 28 days. We hypothesize it is not appropriate to express the data from a known static system as a rate but indicate nominal rates here to test the hypothesis. One sample of BaSO_4_ was left to settle in PSF for nearly 2 years in a 200-mL beaker. The resulting ion concentration after nearly 2 years was identical to the concentration after 7 days. The addition of EDTA, to mimic alkaline earth metal-transporting proteins, only minimally increased BaSO_4_ solubility to 9 mg/L within 28 days (or 0.09%). This is in contrast to recent investigations on Zn-ENM, for which adjustment of the relevant medium was sufficient to induce dissolution, thus better-matching the lack of *in vivo* biopersistence^20^.

We also evaluated the quasi-dynamic dissolution of BaSO_4_ using dialysis^43^. The ion concentration in the receptor medium remained roughly constant in this system: 1.3 mg/L, 1.2 mg/L, 1.1 mg/L, 1.0 mg/L, and 2.0 mg/L on days 1, 2, 3, 4 and 7, respectively. The cumulative dissolution of 0.07% over 7 days and an apparent dissolution rate of k = 0.01 ng/cm²/h remained on the same level as the static solubility system, but below *in vivo* rates. This indicates that an equilibrium Ba concentration of about 1 to 2 mg/L in the pH 4.5 PSF medium is the limiting factor preventing further dissolution.

### Dynamic abiotic dissolution

**Figure 2:**
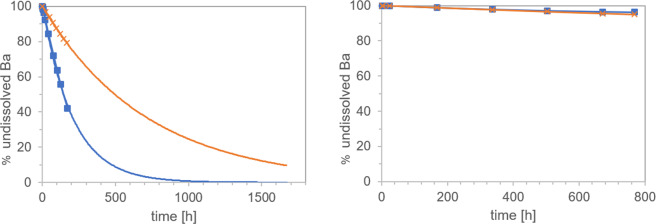
Dissolution kinetics of BaSO_4_ (starting mass, ~1 mg) in pH 4.5 medium, tested by two dynamic dissolution methods: (**A)** flow-through (BASF, blue boxes) or flow-by (Rochester, orange crosses) macrodialysis. (**B)** comparison of both simulant fluids in the flow-through geometry (starting mass, 50 mg): PSF (blue boxes), EU pH4.5 (orange boxes). Note the different ranges of the y-axes.

Motivated by a correlation between the *in vivo* biopersistence of mineral fibers and their abiotic dynamic dissolution rates, two laboratories independently evaluated the dissolution of BaSO_4_ NM-220 using similar macrodialysis systems: one flow-by and one flow-through (Fig. 1). While a flow-by system with the EU pH4.5 medium (composition in Table S1) was used at the University of Rochester, a flow-through system with PSF medium (Table S1) was used at BASF SE. The initial mass (~1 mg BaSO_4_) was the same and the flow rates (2–3 mL/h) were similar. Both labs found that BaSO_4_ NM-220 exhibited a significant dissolution (≥20% over 7 days) under dynamic conditions (Fig. 2A). The dissolution rates and half-times for the two setups do not agree quantitatively despite similar initial mass loadings and flow rates. Exponential fits (Fig. 2A) indicate dissolution t_1/2_ values of 5.9 days (flow-through, pH 4.5 PSF) and 28.9 days (flow-by, EU pH4.5), estimating about 10% uncertainty due to extrapolation of surface areas for the flow-by data. Factors that could slightly impact the comparability of dissolution tests for partially-biosoluble materials such as BaSO_4_ include temperature, different composition of pH4.5 media, flow geometry, membrane pore size, and initial loading. In flow-by geometry, the diffusion of ions from the sample compartment through the membrane into the flow (receptor) compartment could add another rate-limiting step that tends to reduce the apparent dissolution rate as observed from the ion concentrations in the flow compartment.

We replicated the experiment at BASF using EU pH4.5 and the flow-through system, keeping all other parameters unchanged except that the starting mass was increased to 50 mg. The dissolution kinetics in the flow-through setup were identical between the more complex EU pH4.5 and the simpler PSF pH4.5 media over the first few days (Fig. 2B). After 7 days, the dissolution rate in the PSF medium slowed down slightly as compared to the rates in the EU pH4.5 medium. Although it is possible that the phthalates in the PSF acted as ion scavengers – which would increase the solubility limit – PSF seems to slightly favor re-precipitation, thus reducing the apparent dissolution rate. To confirm this hypothesis, we changed the temperature during flow-through dissolution from 37 °C to 4 °C but started with the same initial mass of 1 mg. The apparent dissolution rate was reduced significantly by a factor of 2.5 (data not shown). Also, dissolution in neutral pH medium at 37 °C demonstrated significantly lower rates compared to media with pH 4.5 (Figs. S1 and S3), whereas an omission of organic acid and salts resulted in slight acceleration of apparent dissolution by 7% (data not shown).

Both labs observed that the dissolution kinetics depend on the initial mass of BaSO_4_ (Table 2). Specifically, both labs observed that the Ba ion concentration was limited to a maximum of ~1 mg/L in the eluate from flow-through cells and to ~0.3 mg/L in the dialysate from flow-by cells. This limit is better reflected by the integral of the total mass of ions over the entire duration of the dissolution period, M_ion_/T (Eq. 1a**)**, which turns out to be limited to about 60 µg/day in flow-through and about 15 µg/day in flow-by geometry (Table 2) at the specific flow rates used here. See also Figs. 2B and S2, demonstrating that higher M_O_ leads to system saturation.

**Table 2:** Evaluation of cumulative dissolution of BaSO_4_ in flow cells with pH 4.5 media using flow-through or flow-by methodology.

	M_0_	M_ion_/T	${{\boldsymbol{t}}}_{1/2}$	${{\boldsymbol{k}}}_{{\boldsymbol{diss}}}$	${{\boldsymbol{t}}^{\prime} }_{1/2}^{}$	${{\boldsymbol{b}}}_{{\boldsymbol{diss}}}$
		[µg/d]	[d]	[ng/cm²/h]	[d]	[%/d]
Flow-through, PSF pH4.5	0.17 mg	17.5	2.6	44.7	1.6	43.3
	1 mg	51.1	5.9	10.3	6.8	10.2
	10 mg	53.6	72.1	0.9	78	0.89
	50 mg	58.0	247	0.2	346	0.2
Flow-by, EU pH4.5	0.08 mg	3.9	5	13.8	5.6	12.4
	0.8 mg	13.7	28.9	3.4	25.7	2.7
	8 mg	14.7	72.2	0.32	53.3	1.3

The half-times ${{\boldsymbol{t}}^{\prime} }_{1/2}^{}$ are obtained from *b*_*diss*_ by direct fitting of the decay curve on a semi-log plot using Eq. (2a), whereas the half-times ${{\boldsymbol{t}}}_{1/2}$ are derived via conversion of the cumulative rate *k* by Eq. (2b).

Although the mass loadings in abiotic dissolution experiments are not likely to mimic realistic *in vivo* exposure conditions, an observed solubility limit may be predictive of saturation-related events that occur *in vivo*. This is discussed further below, but first we rationalize the solubility limit by considering the ion sources (by particle dissolution) and ion losses (by flow^36^, flow cell geometries and reprecipitation).

The maximum observed ion concentration in flow-through geometry is close to the pH 4.5 solubility limit observed in the static and quasi-dynamic geometries but is about an order of magnitude higher than in flow-by geometry. The lower threshold of the flow-by system is attributed to the ion concentration in the local vicinity of the particles reaching the pH 4.5 solubility limit. We interpret the data to suggest that upon reaching the pH 4.5 solubility limit locally, ions reprecipitate before the flow removes them. Particle growth in the presence of dissolved ions under supersaturation conditions can lead to the growth of select NPs by the driving force of a lower-energy state of the overall system (Ostwald ripening process). The process matures further when the mobile ionic species (dissolved ions) are exchanged with and precipitated at immobile NPs surfaces and this process is driven by the difference in chemical potential at select NPs surfaces in confined spaces. The overall result can be a particle size increase and various structural and morphological changes (shape, size, crystallinity) of the affected NPs. This phenomenon can indeed be observed in both the flow-through and flow-by systems, i.e., only the measurements at lowest M_0_ (0.17 mg or 0.08 mg, respectively) remain below this limit. We also doubled the flow rate to V = 4 mL/h for M_0_ = 10 mg and observed an increase in M_ion_/T to 102 µg/day, which is roughly twice the observed value at a flow rate of V = 2 mL/h (Table 2). In summary, the cumulative apparent dissolution rates *k* and b (Table 2) scale roughly linearly with the SA/V ratio. Thus, to avoid reaching the solubility limit, either the initial mass can be reduced, or the flow can be increased.

**Figure 3:**
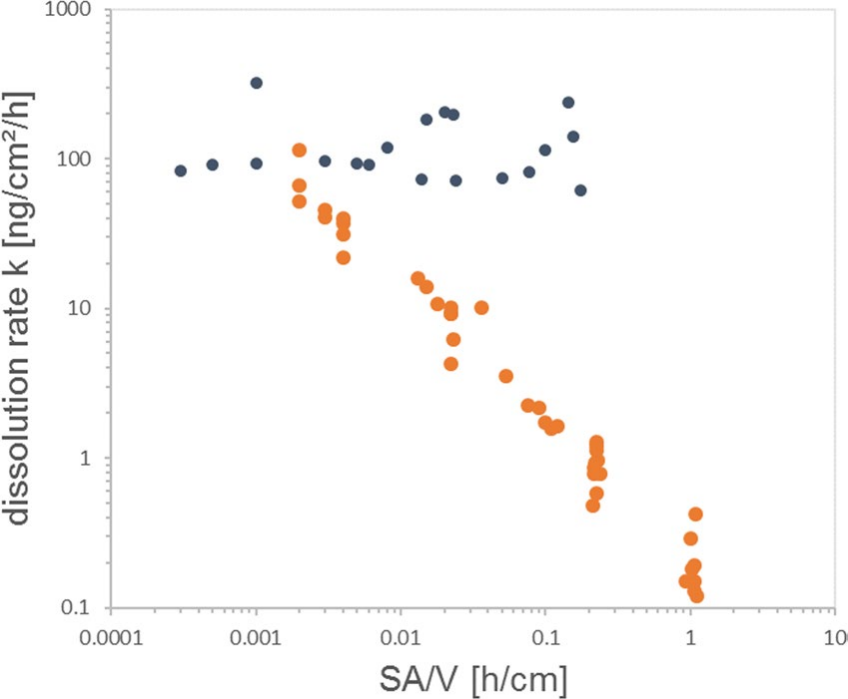
Instantaneous rate evaluation of biodissolution of BaSO_4_ in flow-through cells with pH 4.5 PSF media. Each cloud of stepwise rates stems from separate experiment of initial mass M_0_ and volume flow V. Five experiments for BaSO_4_ (orange) and two for CuO (black). See Table 2 for conventional evaluation (cumulative rates) of the same raw data.

We extensively tested both the reduction of initial mass or the increase of flow independently and in combination and analyzed both cumulative and instantaneous dissolution rates of the identical raw data. If we determine for each sampling interval the instantaneous rates k (in units of ng/cm²/h, Eq. 3) and the instantaneous surface area per volume flow SA/V (in units of h/cm, Eq. 4), hundreds of instantaneous release rates collapse on a single linear relationship, regardless if SA/V was modulated by initial surface area or by flow rate or by gradual dissolution (Fig. 3). The best match of the predicted halftime with the *in vivo* halftime is obtained for SA/V ratios around 0.01 to 0.03 h/cm. To ensure that this unusual observation is not an artifact of the experimental parameters, but truly a material-specific phenomenon, we tested another nanomaterial under identical conditions. We chose 10-nm CuO because it is a benchmark material in the draft OECD guideline on nanomaterial solubility and dissolution^22^ and in the DF4nanoGrouping framework^44^. Like BaSO_4_, no redox processes are involved in CuO dissolution, whereas other benchmark materials might differ by oxidative or reductive dissolution mechanisms^12^. If the CuO dissolution process is mediated by the ENM surface, re-precipitation remains irrelevant, and our calculation is correct, then *k(t)* of CuO should be constant for all *t* until full dissolution. Indeed, the instantaneous dissolution rates *k* of CuO were independent of SA/V across many orders of magnitude (Fig. 3), contrasting to the BaSO_4_ NM-220 behavior. Both CuO and BaSO_4_ do not change their redox state upon dissolution, and are thus non-reactive dissolution processes: in other cases, e.g. metallic Cu, Ag, MoS_2_, CdS etc., the electron exchange with a reaction partner from the surrounding medium may impose a rate-limiting step. In depth work on metal nanoparticles with a scope on the redox reaction forming the soluble product was done in by Gray *et al*. in 2018^12^.

Table 2 also highlights that the half-times ${{\boldsymbol{t}}}_{1/2}$ obtained by direct fitting on a semi-log plot are in close agreement with the half-times ${{\boldsymbol{t}}^{\prime} }_{1/2}^{}$ derived from conversion of the cumulative dissolution rate via Eq. 2a. Of note, the conversion assumes an exponential shape of the decay curve. Only for the highest initial loadings M_0_ ≥ 8 mg, the values disagree significantly, because the saturation processes are reflected by linear (not exponential) kinetics (Fig. S3).

**Figure 4:**
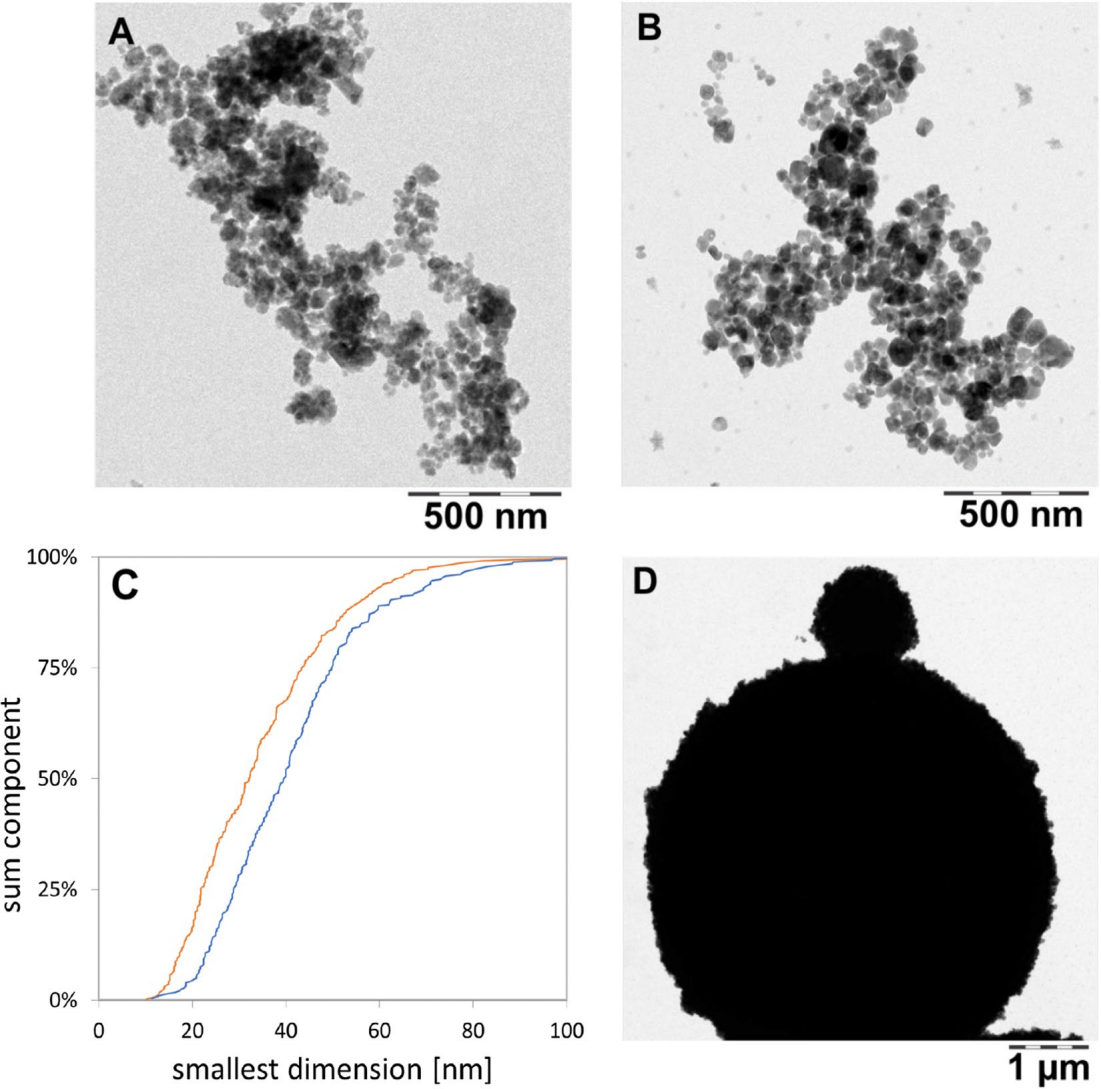
TEM images of BaSO_4_ transferred from remaining solids onto TEM grids (scale bar corresponds to 500 nm for (**A**,**B)** and 1 µm for (**D)**. Panels: A as-produced particles; (**B**,**D)** after 72 hours of treatment in the flow-through cells with PSF. **C** shows the TEM particle size analysis of pristine BaSO_4_ particles (orange) and BaSO_4_ particles after treatment in PSF (blue) (N_A_ = 331; N_B_ = 280).

**Figure 5:**
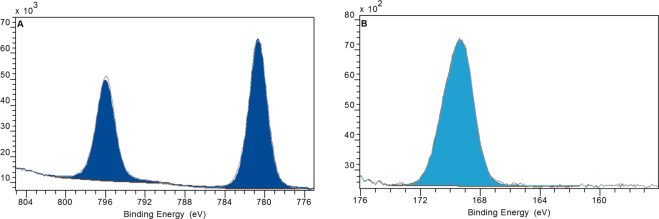
XPS results for BaSO_4_ after flow-through testing in pH 4.5 PSF for 72 h. (**A)** Photoelectron energy line for Ba (3 days). (**B)** Photoelectron energy line for S (2p). The spectra can be fitted quantitatively with the benchmark chemical shifts of BaSO_4_. The elemental composition (Fig. S4) confirms a ratio Ba:S of 1:1.023, all consistent with an identification of the transformation product as BaSO_4_. Data was acquired and averaged on N = 5 measurements.

We also investigated the transformation of the shape and speciation of solids after abiotic testing: We flushed the flow-through cells with water, then opened the cells and rinsed off the remaining solids into a centrifuge vial with a TEM grid at the bottom as described in methodical detail in a recent paper and SI^37^. By centrifugation, all solid >10 nm was spun onto the TEM grid and the supernatant with its buffer salts was discarded. Compared to as-produced BaSO_4_ NM-220 (Fig. 4A), there was a shift towards larger particle diameters (Fig. 4B). Structural rearrangement towards a loss of small radii of curvature are observed (Fig. 4B), and occasionally, very large spherical structures were observed (Fig. 4D). Two different transformation processes are possible reasons for this observation. Ostwald ripening, which is generally explained as a minimization of interfacial energy by an overall increase of the radii of curvature mediated by a minimal solubility of ions first described in^45^. Or secondly, competing intermolecular forces at the particle-particle interface inducing a material transport between particles of different sizes, as deduced from the study of perfluorocarbon blood substitutes^46^. Both processes are driven by the reduction of free energy. When comparing the TEM median particle size before and after continuous flow in PSF (Fig. 4C**)**, a size shift from 32.2 ± 16 nm to 39.9 ± 16.4 nm was measured (by manual evaluation of approximately 300 particles). In contrast, the “shrinking sphere” model^36^ would predict a diameter of 23 nm to match the 60% mass loss that is quantified as dissolved ions in the same experiment. XPS analysis confirmed that the preferred recrystallization species is BaSO_4_ (Figs. 5 and S4), in accord with the *in vivo* EDS observations (see next section).

With the present protocol, the flow conditions are highly controlled, but it is not possible to image the same nanoparticle over time. We also explored an alternative approach, where we repeatedly imaged the same ensemble of nanoparticle very far below the solubility limit at pH 4.5, but without controlling flow. The repeat scan shows that the sphericity of the remaining structures increases at the expense of structures with smaller radius of curvature (Fig. S5), consistent with either of the material transport mechanisms.

### Bioprocessing of BaSO_4_ particles in lung tissue: contributions to overall retention kinetics

**Figure 6:**
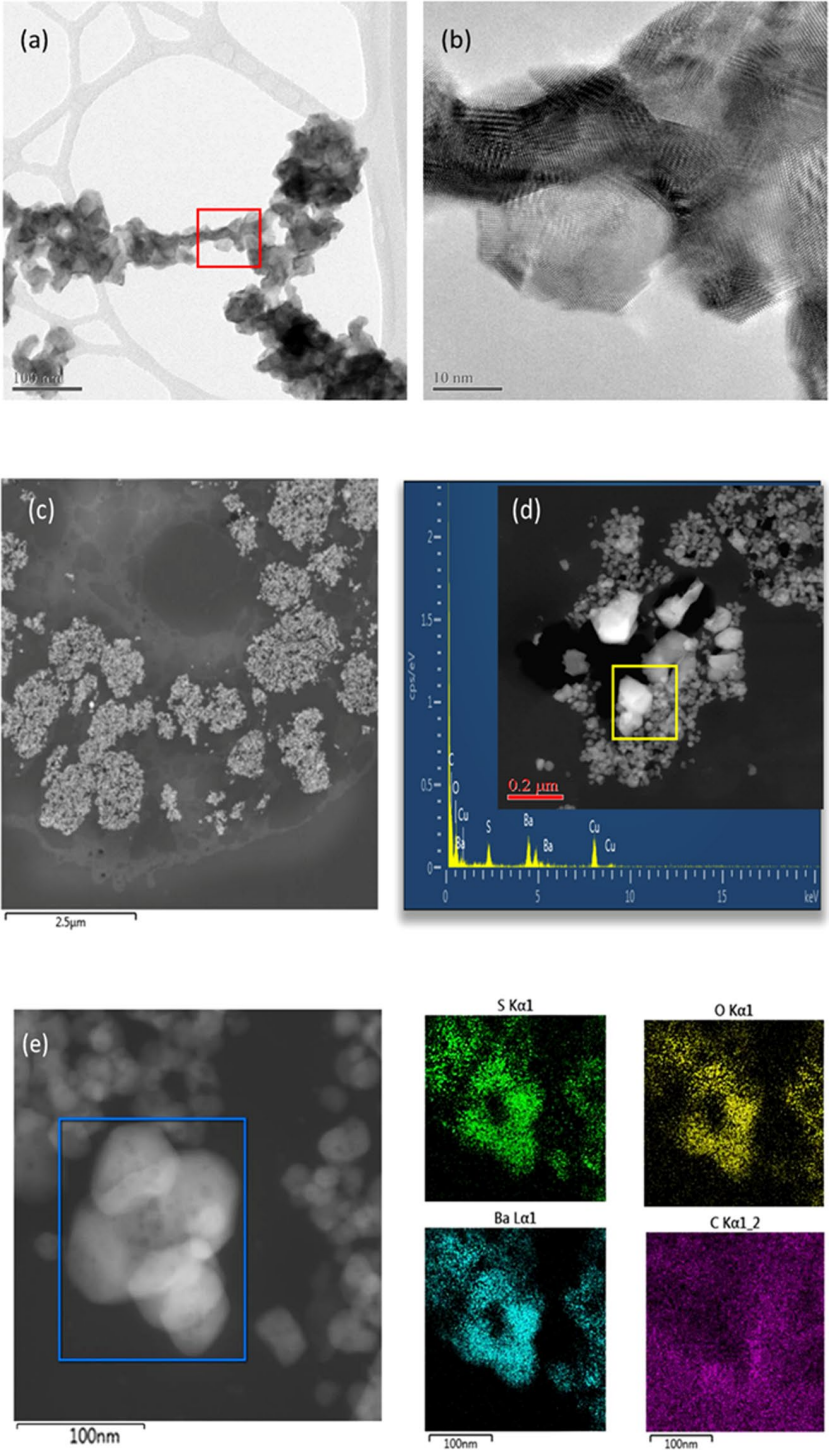
HRTEM/STEM structural characterization of as-produced BaSO_4_ nanoparticles and in rat lung sections. **(a,b)** pristine BaSO_4_ NM-220; **(c)** BaSO_4_ after 12 months inhalation exposure: accumulation in lung macrophage; **(d)** Transformation of BaSO_4_ in macrophage showing particle growth with crystalline facets. **(e)** HRSTEM of recrystallized BaSO_4_ in macrophage with corresponding high-resolution EDS mapping for S, Ba, O and C.

Long-term inhalation exposures (12–24 months) to BaSO_4_ NM-220 (Fig. 6a,b) at a high aerosol concentration (50 mg/m³) resulted in significant accumulation of BaSO_4_ in lung macrophages (Fig. 6c,d). The retention of BaSO_4_ particles in the lung had reached a maximum at ~12 months of exposure. At 12 months of continued inhalation exposure, the retained dose of BaSO_4_ did not increase further, despite continued exposure. It appears that the continued pulmonary deposition of inhaled BaSO_4_ was counterbalanced by removal (dissolution of the nanoparticles due to shedding of ions from the particle surfaces and elimination from the lung). The retained Ba in the lung was, thus, at an equilibrium between 12 and 24 months of exposure. The lung macrophages (Fig. 6c) contained nanoparticles that were identified with high resolution EDS to correspond to BaSO_4_ (Fig. 6e). The spacing or relative distance between BaSO_4_ particles inside the macrophages was rather small, with many particles seeded side-by-side (Fig. 6c–e). Surprisingly, the particle size distribution of BaSO_4_ inside macrophages was significantly larger as compared with the parent material (Fig. 6a,b). This indicates that Ba and SO_4_ ion concentrations inside the macrophage environment that contained many densely packed BaSO_4_ particles, approached supersaturation conditions following the long-term inhalation exposure at a very high aerosol concentration (50 mg/m^3^). This resulted in the transformation and recrystallization of particles inside the lung macrophages. Material transport between particles had also occurred, whereby smaller particles dissolved faster, and the released ions were not removed, but rather deposited onto neighboring particle surfaces, allowing selective particle growth to take place (Fig. 6d,e). This is the first documentation of an inter-particle material transport mechanism of nanoparticles after uptake *in vivo*. In addition, BaSO_4_ particles exhibited a greater size range (from sub-nano to micron scale) as compared with that of the starting materials which is in good agreement with observations from the abiotic flow through cells. Transformation of BaSO_4_ involved a particle size effect and a recrystallization of the particles which was controlled by the saturation and supersaturation conditions in the macrophage microenvironment. The shedding of ions drove not only the *in vivo* dissolution kinetics, but also controlled the shape, morphology, and size distribution of retained BaSO_4_ in the lung. This indicates that BaSO_4_ undergoes transformation mediated by non-equilibrium dissolution (in line with the observed incorporation of Ba in bones within days after intratracheal instillation) and recrystallization, thus modulating the overall biopersistence of the particles. Not all macrophages contain copious amounts of densely packed BaSO_4_ and it is important to point out that each macrophage represents a unique system where dissolution and supersaturation conditions are subject to the nanoparticle accumulation rate. Only when enough BaSO_4_ nanoparticles collect inside a macrophage can transformation occur.

## Discussion

We posit that the measurement of dynamic particle dissolution should be an important element of predictive toxicity testing, i.e., the determination of dissolution *rates* in abiotic systems as opposed to static *solubility*. The *solubility* categorizes BaSO_4_ (including the specific NM-220 grade) as poorly-soluble in water. However, solubility in water does not reflect *in vivo* reality in terms of 1) ongoing dynamic processes and 2) composition and pH of physiological fluids. The *dissolution rates* in physiological media better reflect an important component of *in vivo* particle clearance, considering that both absorptive chemical and physical clearance mechanisms are always working in tandem to affect total particle clearance (see below). Dissolution rates determined in appropriately-designed *abiotic* systems will be useful for grouping and classification of ENMs. From these predictive testing results, we also gain insight into the mechanisms that underlie biosolubility, which may explain experimental findings, e.g., the incorporation of Ba^2+^ in bones (as Ca^2+^ analogue).

Concerning the methodology, a solubility limit of ~100 mg/L has been proposed for testing strategies^30,47^ and grouping frameworks^13^ to define readily-soluble particles that would quickly lose their particulate nature. A currently developing OECD guideline describes a “screening test” that fulfills the requirements for Tier 1 grouping frameworks^22^. For purpose of initial screening of equilibrium *solubility*, the test could be performed in water, and then in the most relevant medium for exposure-specific testing^20^. For ENMs with solubility limits below 100 mg/L, for hazard assessment of innovative nanomaterials, or for endpoint-specific grouping and read-across between nanoforms, flow-by or flow-through dynamic dissolution (both are continuous flow systems^36^) in relevant media can offer predictivity of *in vivo* dissolution behavior^10^, but not necessarily total *in vivo* clearance rates.

In order to best model the contribution of dissolution to *in vivo* clearance, it makes good sense to focus on the intraphagolysosomal environment at pH 4.5 (although dissolution in extracellular fluid (at neutral pH) could also be considered and included in kinetic model equations). Additional information about the impact of different flow rates and starting masses would provide insight regarding saturation, which could be predictive of *in vivo* events. The selected methods for ion analysis should ideally have a limit of detection of 10 µg/L or below for the target analyte. Methods for morphological analysis of remaining solids require sample preparation, which was developed here based on the NanoDefine D6.3 protocol for TEM analysis, using centrifugal pelleting of remaining solids onto a TEM grid (which inherently is a purification from dissolved species). To assess the extent of transformation, we recommend TEM-based morphological and size distribution analyses (N»100), with optional confirmation of chemical speciation, e.g., via SAD or XPS or XANES. For materials other than BaSO_4_, more complex re-speciations must be expected, e.g. Ag sulfidation, Cu oxidation^48,49^, CeO_2_ re-speciation to CePO_3_ needles^29^). Reprecipitation in flow-cells has been observed frequently during dissolution testing of stone wool mineral fibers, where especially Si tends to reprecipitate as gel on the surface of the fibers^50–53^. Most interestingly, a faster flow (lower SA/V ratio) is known to suppress gel formation and to increase the apparent stone wool dissolution rate^54^. These reports are analogous to our findings with BaSO_4_.

Dissolution should be expressed in terms of rate (e.g., k in units of ng/cm²/h or %/day) for which ample literature on dusts exists^10^. This approach challenges the cutoffs for categorization and grouping of ENMs, typically expressed in % dissolved or mg/L concentrations. BaSO_4_ is clearly “insoluble” in mg/L metrics as determined using static systems but is correctly predicted to dissolve *in vivo* by the dynamic dissolution methods.

The findings from these studies could be used to propose alternative categories for grouping approaches of ENMs. For the rat, lung retention half-times (*t*_1/2_) for ‘poorly-soluble low-toxicity’ particles (PSLTs) are ~70 days and, by definition, reflect mechanical, macrophage-mediated clearance, generally following first-order kinetics^10^. Half-time and rate constants are inversely related to each other via Eq. (2). Using this equation to determine a rate constant for PSLTs yields ~0.01/day or roughly 1% of starting material – as expressed using any metric of choice – per day for the mechanical component of clearance. Knowing that total lung particle clearance reflects the sum of mechanical and dissolution clearance, one can derive groups of dissolution clearance rates such that faster rates would indicate readily- or partially-biosoluble particles. In a previous paper we applied the continuous flow system to 24 different ENMs, and suggested decadic ranges of the dissolution rates between <1 ng/cm²/h (insignificant dissolution – also asbestos falls into this group) and >100 ng/cm²/h (half-times on the order of 1 day)^37^. Expressing dissolution rates in percent per day enables comparisons to and predictions of *in vivo* clearance rates.

The preceding discussion is based on results from studies that were conducted in rats but could be adapted to human hazard characterization via the use of human-specific rate constants or retention half-times. While mechanical clearance rates exhibit species specificity and also be impacted by inflammatory responses, it is predicted that dissolution rates are similar between humans and rodents^10^. The method could be used to enhance the DF4nanoGrouping^13^, and to implement the ECHA grouping guidance^55^ pending further validation by more varied case studies as proposed elsewhere^37^.

With regard specifically to BaSO_4_ pulmonary clearance and transformation, Konduru and colleagues reported that intratracheally instilled ^131^BaSO_4_ NM-220 exhibited a lung retention half-time of 9.6 days in rats and that ^131^Ba was incorporated into the bones, suggesting nanoparticle dissolution and/or extrapulmonary translocation^3^. A subsequent 90-day inhalation study in rats with aerosolized BaSO_4_ of the same grade (50 mg/m^3^, full physicochemical equivalence to NM-220^4^) showed the gradual accumulation of Ba in lung tissue during exposure followed by steady clearance over a 90-day post-exposure period, with a reported retention half-time of 56 days, indicative of low *in vivo* solubility of BaSO_4_ affecting its overall lung clearance. A two-year rat inhalation study in rats with BaSO_4_ NM-220 (50 mg/m^3^) confirmed a steady increase of retained Ba in the lung up to one year of exposure, with no further increase during subsequent continued exposure up to two years^5^. The equilibrium lung burden of Ba over the exposure period (12–24 months) is explained by the fact that the daily deposited dose in the lung is equal to the amount being cleared daily, i.e., deposition and clearance rates are in equilibrium. Knowing the BaSO_4_ aerosol characteristics (mass median aerodynamic diameter, geometric standard deviation, exposure concentration) and exposure duration, the daily deposition rate can be estimated using the MPPD model for rats with body weight-adjusted respiratory parameters, which results in a daily BaSO_4_ lung clearance rate of 0.0154% of the daily deposited dose. This is equivalent to a retention half-time of 45 days (Eq. 2a). Since the lung clearance rate for biosoluble particles is the sum of mechanical and dissolution clearance rates, the difference between the normal rat clearance rate for PSLT particles (0.01/day) and the observed clearance rate in the equilibrium phase (0.0154/day) is the BaSO_4_ *in vivo* dissolution clearance rate (0.0054/day; t_1/2_ = 128 d) (Table 3). The available data show that, for acute exposures, rapid clearance of BaSO_4_ occurs and that dissolution contributes significantly to the total clearance. Following subchronic or chronic exposures, total lung clearance is slower, but is nevertheless faster than mechanical clearance alone. Of note is that the predicted dissolution rates and associated half-times for the 90-day and two-year studies are prolonged as compared to acute exposures, suggesting a saturation event. Both the fast, short-term *in vivo* dissolution at a lower ‘dose’ and the saturation at higher ‘doses’ were predicted by the abiotic assay (Table 2). The bioavailability of ionic Ba could predict secondary organ uptake with the caveat that there could be macromolecule binding events that might limit the clearance of Ba.

**Table 3:** Pulmonary dissolution rates and associated half-times for BaSO_4_ derived from acute and repeated rat exposure studies.

Exposure duration	Particle type and method of delivery	Total Retention *t*_*1/2*_ (d)	${b}_{tot}$ (d^−1^)	Dissolution Retention *t*_1/2_ (d)	${b}_{diss}$ (d^−1^)
---- ^(3)^	^131^BaSO_4_ NM-220 intratracheal instillation	9.6	7.23%	11.1	6.23%
90 days ^(4)^	BaSO_4_ NM-220 nose-only inhalation	56	1.24%	289	0.24%
2 years ^(5)^	BaSO_4_ NM-220 head-nose inhalation	45*	1.54%	128	0.54%

^*^At steady-state.

Depending on the initial loading, flow rate and flow cell geometry, the b_diss_ in the abiotic test ranges from 0.2% at strong saturation to 43% well below saturation, and abiotic dissolution half-times range from 350 days to 2 days (Table 2). The range of the half-time and rate values includes that which was found in the different *in vivo* studies. One might interpret that the intratracheal instillation study induced no or only mild saturation (locally), whereas the 90-day and two-year inhalation studies induced significant saturation, consistent with the morphological observations (Fig. 6).

The collective *in vivo* findings and those from the present dissolution studies suggest that Ba ions dissolved from lung-deposited particles – as opposed to the particles themselves – and were transported throughout the body and incorporated in bone epiphysi^56–58^. Furthermore, the long-term inhalation study results can be explained by a phenomenon whereby bone tissue – with its limited capacity and varying demand for bivalent cations over time – was saturated^59^, after which the net transport of Ba from lungs to bone decreased and, ultimately, the accumulation of Ba in the lungs increased. Within the (local) environment of the lungs, the ion removal rate depends on many factors, e.g., binding to biomolecules, that may affect the clearance rate.

Whether there is a specific or non-specific transport mechanism of Ba ions from the lung or a key trigger whose signaling results in the reduced removal of Ba from the lung remains to be elucidated. The measurement of Ba blood levels might help to shed light onto these questions. In addition, local clearance mechanisms in the lungs, such as mucociliary clearance and the clearance by alveolar macrophages, might be prolonged. We qualitatively observed significant accumulation of BaSO_4_ in rat lung macrophages exposed for 12 months or longer to BaSO_4_. Of note, the acidic pH of the macrophage lysosome is essential to BaSO_4_ dissolution, which is very significantly reduced at pH 7.4 (Fig. S1), by ~35% as compared to pH 4.5. Furthermore, removing the organics from the PSF pH 4.5 medium results in a significant decrease in dissolution (Fig. S3) and thus points to the ion scavenging effect of organic acids in lysosomal fluids. Taken together, uptake in macrophages and active transport of the ions are most likely steps in the clearance pathway, but dissolution in the neutral lining fluid may also contribute to total *in vivo* clearance.

Since the time-resolved abiotic dissolution shows that saturation conditions are reached, and furthermore crystalline particle growth was observed *in vivo* as well as in abiotic conditions, the structural transformation process are best described as Ostwald ripening. Once the net transport ceases, accumulation entails supersaturation conditions leading to the Ostwald ripening in macrophages that have accumulated BaSO_4_ particles in phagolysosomes. Since each macrophage harbors unique concentrations of BaSO_4_ particles, there are just as many systems (local supersaturation) to be considered. In this concept, the structure formation process is a self-catalyzing phenomenon: once the local ion concentration exceeds the solubility, triggering particle growth, the specific surface area of the deposited particles decreases, thus slowing dissolution until equilibrium is reached between removal via dissolution and addition by deposition. Further evidence for *in vivo* Ostwald ripening of inhaled BaSO_4_ particles in lung tissue was recently presented^60^. Families of (nano)forms that share each one substance but differ in sizes, coatings or shapes, have been assessed by the same methodology for dissolution and transformation^37^. The BaSO_4_ dissolution rate is intermediate in comparison, and materials such as amorphous silica show related reprecipitation phenomena, albeit at slower rate^37^.

Although the present study was designed to rationalize the clearance of BaSO_4_ after inhalation, we note that the same concepts of local supersaturation (reaching the solubility limit of the specific ion in the lung medium) may be relevant to understand biokinetics after any other uptake routes. Accumulation of Ba in the lungs was reported after IV injection^3,61^: Giese 1934 and 1935 found after IV injection of BaSO_4_ deposition in bone marrow, liver, spleen and lungs. Konduru *et al*. found 20% of the administered dose in lungs at 2 days post IV injection^3^. Ba ions are likely to have precipitated, similar to the observation via HRTEM of newly formed Ce-containing (nano)particles in the liver^43,62^. Huston *et al*. observed the formation of “refractile masses” after instillation of a Ba containing solution Veriopaque, which was accompanied by inhibited removal of Ba by macrophages.

Ba^2+^ ions elicit systemic toxicity mainly via hypokalemia that is caused by the blocking of rectifying potassium channels in many cell types^63^. Although these effects do not necessarily require cellular uptake, it should be pointed out that Ba^2+^ ions, similar to Sr^2+^ ions, are capable of permeating specific Ca channels as well as non-selective cation channels of the cell membrane^64–66^, and some of these channels have been shown to be essential for macrophage function^67–69^. Ba^2+^ ions may also be actively transported against an electrochemical gradient by Ca^2+^ ATPases^70^. Once inside a cell, Ba^2+^ ions may further pass to the different cell organelles such as endoplasmic reticulum, mitochondria, and lysosomes^71^. Thus, Ba^2+^ ions dissolving from BaSO_4_ nanoparticles may distribute across membranes between subcellular compartments with the net fluxes being determined by electrochemical driving forces: of note, phagolysosomes are acidified by an active transport of H^+^ ions carried out by V-ATPases^69^, resulting in a positive potential of approximately 30 mV. This potential may act as an outward driving force for Ba^2+^ ions, while at the same time creating an inward driving force for chloride ions, e.g., via CLC channels^68,69^. Although not yet proven it is tempting to speculate that these processes may be involved in fostering dissolution and/or recrystallization processes of BaSO_4_ nanoparticles trapped in a macrophage’s phagolysosome.

## Conclusion

Our results confirmed the previous findings that prediction of dissolution rates requires the use of relevant biological/physiological fluids rather than water^20^. The methodologies described herein for measuring abiotic dynamic particle dissolution and transformation involve a number of improvements:

- The use of continuous flow, rather than static incubation;
- The integrated assessment of residual solids with respect to transformations of shape, size distribution, and crystallinity by protocols for preparation, analysis and statistical image analysis, using TEM, optionally supported by XPS and EDX.
- The ready comparison, using the same experimental system, to compare to rapidly- and poorly-soluble benchmark particles, for grouping purposes as demonstrated elsewhere^37^;
- Ability to predict *in vivo* dissolution rates (with the acknowledged limitation that dissolved ions could be retained in tissues via binding to other molecules);
- The observation of dependence on initial loading mass could be useful for estimating *in vivo* solubility limits and, thus, provide insight regarding supersaturation that would impact total clearance rates.

Specifically, to BaSO_4_ we propose that the unusual biokinetics of the long-term, high concentration BaSO_4_ rat inhalation studies indicate a) the release of Ba ions via *in vivo* dissolution of phagocytosed particles, with transport to and uptake into the bone and b) recrystallization in lungs as additional transformation process that modifies ENM lung retention. The process is a self-catalyzing phenomenon as the specific surface area of the transforming particles decreases, thus slowing down dissolution. Especially the second year of the two-year inhalation study was, thus, conducted at significant saturation. Control measurements on CuO demonstrated that Ostwald ripening and supersaturation phenomena are not a methodical artifact, but characteristic of the BaSO_4_ properties, and were reproducible in two labs and different lysosomal simulants. The rates and the transformation and the Ba speciation were verified *in vivo*, with the only limitation that *in vivo* processing resulted in less sphericity and more crystalline facets. The dynamic dissolution results thus qualitatively predicted the *in vivo* BaSO_4_ dissolution, as well as the concentration-dependent Ostwald ripening process observed within the rat lung.

## Supplementary information

Supplementary Information.

## Data Availability

The test material BaSO_4_ NM-220 is available from the OECD sponsorship repository at Fraunhofer Institute, Schmallenberg. The same-grade-later-batch used for the long-term inhalation study is available as JRCNM50001a in the frame of the PATROLS project from the JRC repository, Ispra.
