# Author Correction: Predicting dissolution and transformation of inhaled nanoparticles in the lung using abiotic flow cells: The case of barium sulphate

**DOI:** 10.1038/s41598-021-82185-5

**Published:** 2021-04-19

**Authors:** Johannes G. Keller, Uschi M. Graham, Johanna Koltermann-Jülly, Robert Gelein, Lan Ma-Hock, Robert Landsiedel, Martin Wiemann, Günter Oberdörster, Alison Elder, Wendel Wohlleben

**Affiliations:** 1 Department Experimental Toxicology and Ecology and Department Material Physics, BASF SE, 67056 Ludwigshafen, Germany; 2 Institute of Pharmacy, Faculty of Biology, Chemistry & Pharmacy, Freie Universität Berlin, 14195 Berlin, Germany; 3 National Institute of Occupational Safety and Health, Cincinnati, Ohio 45226 USA; 4 Biopharmaceutics and Pharmaceutical Technology, Saarland University, 66123 Saarbrücken, Germany; 5 IBE R&D Institute for Lung Health gGmbH, Mendelstr. 11, 48149 Münster, Germany; 6 University of Rochester Medical Center, Rochester, New York USA

Correction to: *Scientific Reports* https://doi.org/10.1038/s41598-019-56872-3, published online 16 January 2020

This Article contains errors. In the Methods section, under subheading ‘Flow-through abiotic dissolution and transformation’,

“For the lower flow rate, this corresponds to a ratio, SA/V = 0.02 h/cm”.

should read:

“For the lower fowl rate, this corresponds to a ratio, SA/V = 0.02 h/µm”.

In the Results section, under subheading ‘Dynamic abiotic dissolution’,

“If we determine for each sampling interval the instantaneous rates k (in units of ng/cm^2^/h, Eq. 3) and the instantaneous surface area per volume flow SA/V (in units of h/cm, Eq. 4), hundreds of instantaneous release rates collapse on a single linear relationship, regardless if SA/V was modulated by initial surface area or by flow rate or by gradual dissolution (Fig. 3)”.

should read:

“If we determine for each sampling interval the instantaneous rates k (in units of ng/cm^2^/h, Eq. 3) and the instantaneous surface area per volume flow SA/V (in units of h/µm, Eq. 4), hundreds of instantaneous release rates collapse on a single linear relationship, regardless if SA/V was modulated by initial surface area or by flow rate or by gradual dissolution (Fig. 3)”.

And,

“The best match of the predicted halftime with the *in vivo* halftime is obtained for SA/V ratios around 0.01 to 0.03 h/cm”.

should read:

“The best match of the predicted halftime with the *in vivo* halftime is obtained for SA/V ratios around 0.01 to 0.03 h/µm”.

Furthermore, the x-axis of Figure 3 is incorrectly labeled as “SA/V h/cm”, whereas the correct unit is “SA/V h/μm”. The correct Figure 3 appears below as Figure 1.

**Figure 1:**
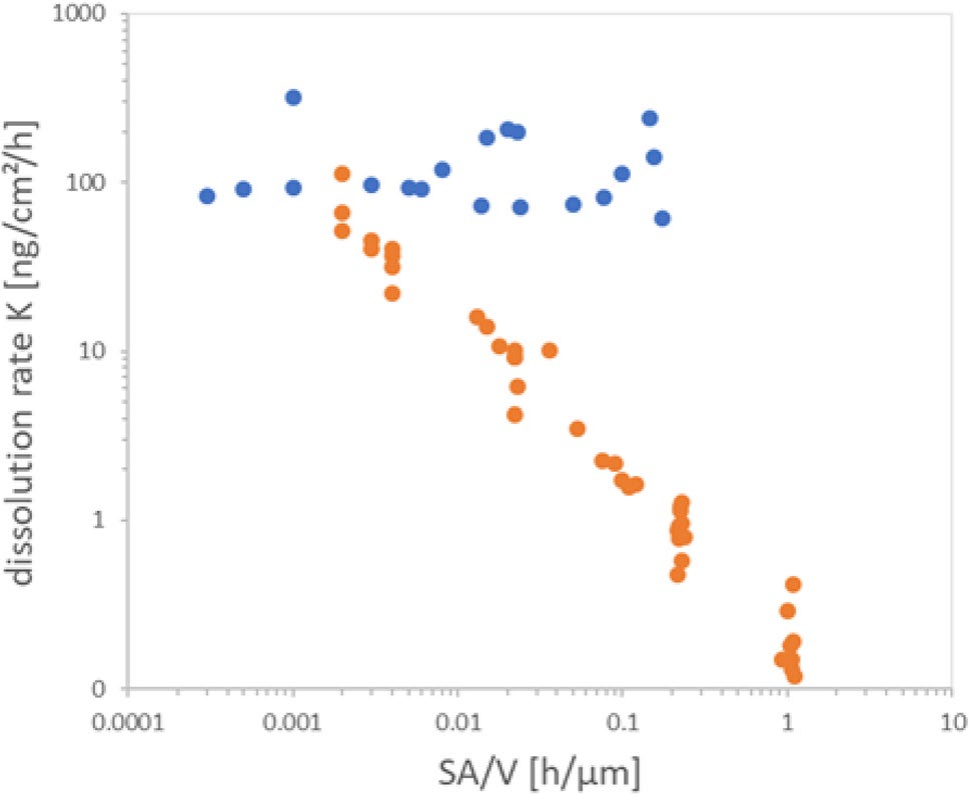
Instantaneous rate evaluation of biodissolution of BaSO_4_ in flow-through cells with pH 4.5 PSF media. Each cloud of stepwise rates stems from separate experiment of initial mass M_0_ and volume flow V. Five experiments for BaSO_4_ (orange) and two for CuO (black). See Table 2 for conventional evaluation (cumulative rates) of the same raw data.

