# Supplementary material for: Predicting dissolution and transformation of inhaled nanoparticles in the lung using abiotic flow cells: The case of barium sulfate
Source: Sci Rep. 2020 Jan 16;10:458. doi: 10.1038/s41598-019-56872-3 (PMC6965653; doi:10.1038/s41598-019-56872-3)
Supplement: Supplementary file 1 — Supplementary Information. [file 41598_2019_56872_MOESM1_ESM.pdf]

Supplementary Information

Predicting dissolution and transformation of inhaled nanoparticles in the lung using abiotic flow cells: The case of barium sulfate

Johannes G. Keller[1]†, Uschi Graham[2]†, Johanna Koltermann-Jülly[1], Robert Gelein[3], Lan Ma-Hock[1], Robert Landsiedel[1], Martin Wiemann[3], Günter Oberdörster[3], Alison Elder[3]*, Wendel Wohlleben[1]*

Affiliations:

† equal contribution

* Correspondence: alison_elder@urmc.rochester.edu; wendel.wohlleben@basf.com

[1] BASF SE, Dept. Experimental Toxicology and Ecology, and Dept. Material Physics, 67056 Ludwigshafen, Germany; johanna.koltermann-juelly@basf.com, johannes-georg.keller@basf.com, lan.ma-hock@basf.com, robert.landsiedel@basf.com, wendel.wohlleben@basf.com

[2] National Institute of Occupational Safety and Health, Cincinnati, Ohio, 45226, USA; graham@topasol.com

[3] Biopharmaceutics and Pharmaceutical Technology, Saarland University, 66123 Saarbrücken, Germany

[4] IBE R&D Institute for Lung Health gGmbH, Mendelstr. 11, 48149 Münster, Germany; martin.wiemann@ibe-ms.de

[5] University of Rochester Medical Center, Rochester, New York, USA; alison_elder@urmc.rochester.edu, gunter_oberdorster@urmc.rochester.edu

Detailed Methods: composition of the buffers

We employed two different media with pH 4.5, both simulating the phagolysosomal interior and the hereby associated processing of phagocytosed particulate matter by alveolar macrophages.[1, 2]

The composition of Phagolysosomal simulant fluid (PSF) is:[3] sodium phosphate dibasic anhydrous (Na_2HPO_4) 142 mg/L; sodium chloride (NaCl) 6650 mg/L; sodium sulfate anhydrous (Na_2SO_4) 71 mg/L; calcium chloride dihydrate ($CaCl_2$ $2H_2O$) 29 mg/L; glycine ($C_2H_5NO_2$) 450 mg/L (as representative of organic acids); potassium hydrogen phthalate (1-(HO_2C)–2-(CO_2K)–C_6H_4) 4085 mg/L; alkylbenzyldimethylammonium chloride (ABDC) 50 ppm (added as an antifungal agent). The pH was adjusted to 4.55 using 1 M NaOH (Bernd Kraft). The composition of EU pH4.5 in comparison contains a richer mix of organic acids and different biocides. However, pH was checked to be stable in eluates of both simulants.

Table S1: Composition of lysosomal simulant fluids (in mg/L)

**Substance**	**PSF pH4.5**	**EU pH4.5**
NaCl	6650	7120
Na_2HPO_4	142	148
Na_2SO_4	71	79
$CaCl_2$ $2H_2O$	29	29
$MgCl_2.6H_2O$		211
KH-Phthalate	4085	0
Na_3Citrate.$2H_2O$		153
Na_2Tartrate.$2H_2O$		180
90% lactic acid		235
NaPyruvate		201
Glycine	450	118
HCO_3^-		1416
alkylbenzyldimethylammonium chloride (ABDC)	50	
H_2CO (Formaldehyde)		799
CH_3OH		270

Detailed Methods: Solubility in phagolysosomal simulant fluid under static conditions

Nano-scaled $BaSO_4$ (NM-220) was suspended either in 200 mL ultrapure water or in 200 mL phagolysosomal simulant fluid (PSF) at a concentration of 10 mg/mL. The $BaSO_4$ suspensions were incubated for 7 or 28 days at 37 °C under continuous stirring (300 rpm). In a second series of experiments EDTA-Na_2 (Sigma Aldrich, 20 mg/mL) was added to the $BaSO_4$-PSF-suspension. EDTA mimicked alkaline earth metal-transporting proteins. Subsequent to the incubation period, the remaining particulate matter was separated from the ion solution using ultracentrifugation at 67,000 ×g for 2 h (Beckman L8-70M ultracentrifuge). From preliminary work, it is known that this material, with its given density, is completely removed from the supernatant at these conditions. The Ba concentration in the supernatants was analyzed by inductively coupled plasma mass spectrometry (ICP-MS). The limit of detection was 0.1 ppm, corresponding to 0.001 % dissolution.

Detailed Methods: Solubility under quasi-dynamic conditions

$BaSO_4$ nanoparticles suspended in PSF (10 mg/mL) were injected into a dialysis cassette (Thermo Scientific). This device was composed of a sealed sample chamber (sample volume 2 mL) enclosed at two sites by dialysis membranes (7 kDa cutoff). The dialysis cassette, harboring the $BaSO_4$ suspension, was placed horizontally (to minimize pelleting) into a glass vessel filled with 200 mL receptor medium (also PSF). This system was kept at 37 °C for 7 days under continuous stirring of the receptor medium (300 rpm). During this incubation time the receptor medium was exchanged completely every 24 h on working days. To minimize evaporation, the glass vessel was closed with parafilm. The ionic Ba content of each receptor medium sample was quantified by ICP-MS.

Additional results

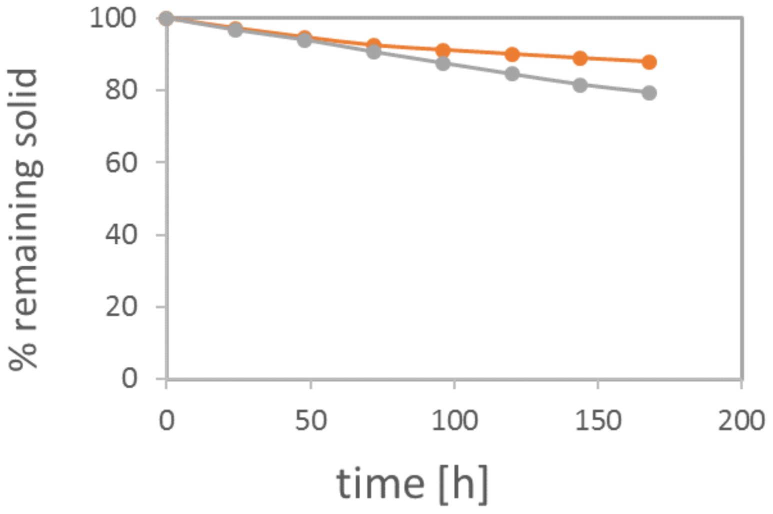

Figure S1 Time dependent dissolution of $BaSO_4$ in pH neutral medium compared to EU pH4.5 in the flow-by system. Orange at pH 7.4 and gray at pH 4.5 (37 °C; starting mass, 1.05 mg for PSF, 0.79 mg for EU pH4.5).

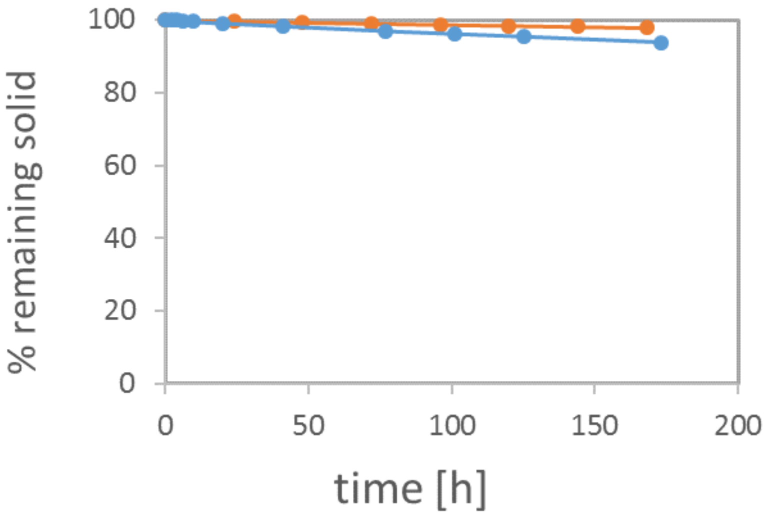

Figure S2 Dissolution of 10 mg $BaSO_4$ NM-220 in PSF measured in flow-through system (blue) and EU pH4.5 in the flow-by system (orange).

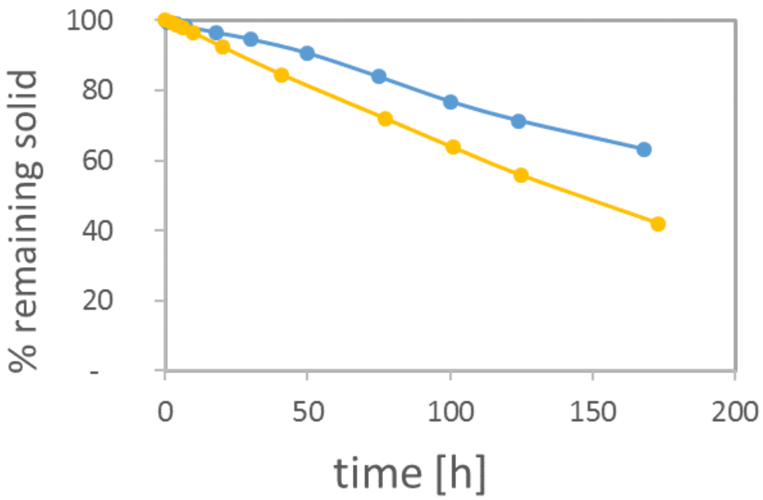

Figure S3 Time dependent dissolution of $BaSO_4$ in deionized water pH 7.0 compared to PSF pH 4.5 in the flow-through system. Blue deionized water at pH 7.0 and yellow PSF at pH 4.5

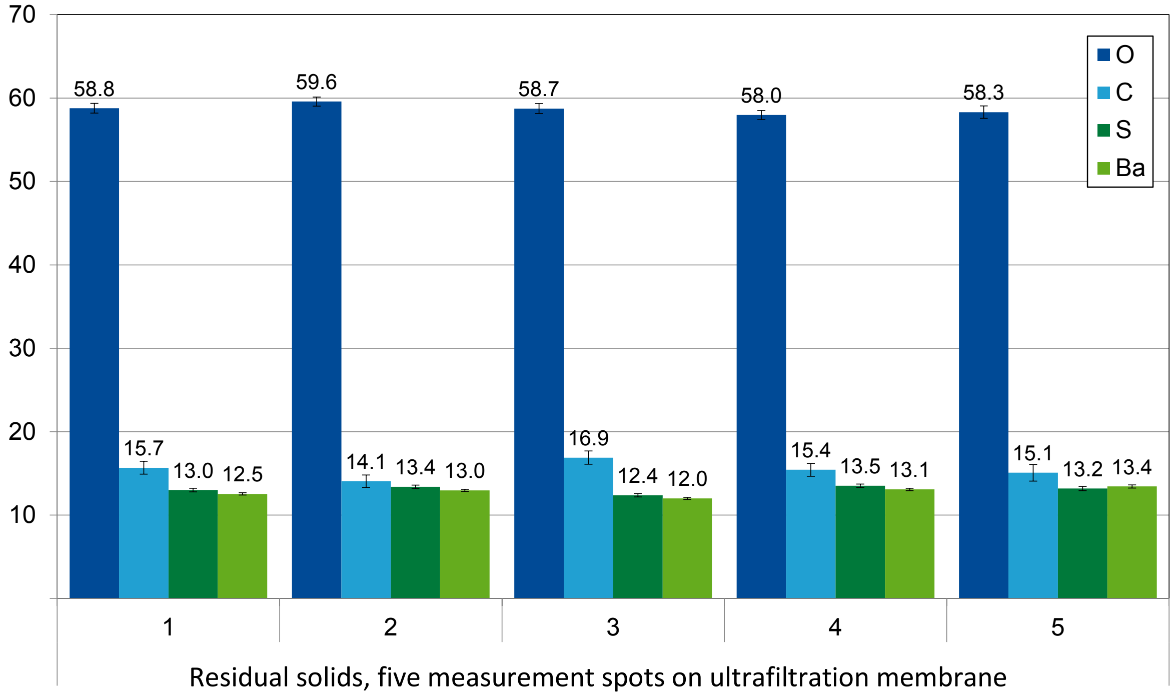

Figure S4 XPS elemental composition (in atom-%) of the residual particles after flow-through testing at V=2 mL/h in pH 4.5 PSF for 72 h. For statistical evidence the results from five measurement spots were measured and compared.

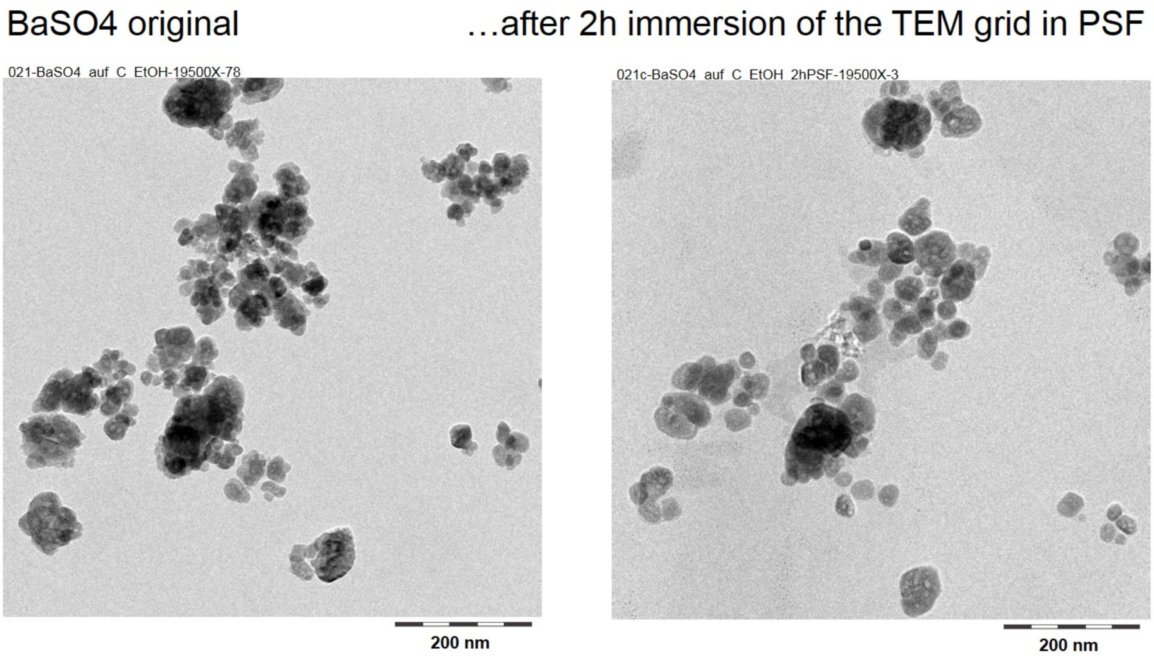

Figure S5 In situ tracking of $BaSO_4$ morphological changes: particles were wetted by ethanol, then immediately dip-coated onto a TEM grid with position markers. The grid was transferred to vacuum and a scan of the 'original' material was acquired. The grid was recovered from vacuum and immersed in PSF pH 4.5 for 2 h, rinsed with water, and a scan at the same position was repeated. This procedure does not remove ions by enforced flow but operates at total solids of less than ng/L, hence far below the equilibrium. The repeat scan shows that the sphericity of the remaining structures increases at the expense of structures with smaller radius of curvature. It appears that 'sphericity' is not seen *in vivo*, where more crystalline phases with flat surfaces are present with Ostwald ripening.

References in SI:

1. Nti: PD ISO/TR 19057:2017 - Nanotechnologies. Use and application of acellular in vitro tests and methodologies to assess nanomaterial biodurability. In: *Nanotechnologies Use and application of acellular in vitro tests and methodologies to assess nanomaterial biodurability*2017.
2. Oberdörster G, Kuhlbusch TAJ. In vivo effects: Methodologies and biokinetics of inhaled nanomaterials. NanoImpact. 2018;10 Supplement C:38-60; doi: https://doi.org/10.1016/j.impact.2017.10.007. http://www.sciencedirect.com/science/article/pii/S2452074817300629.
3. Stefaniak AB, Guilmette RA, Day GA, Hoover MD, Breysse PN, Scripsick RC. Characterization of phagolysosomal simulant fluid for study of beryllium aerosol particle dissolution. Toxicology in Vitro. 2005;19 1:123-34; doi: 10.1016/j.tiv.2004.08.001. http://www.sciencedirect.com/science/article/pii/S0887233304001250.
